# A Comprehensive Investigation of the Potential Role of Lipoproteins and Metabolite Profile as Biomarkers of Alzheimer's Disease Compared to the Known CSF Biomarkers

**DOI:** 10.1155/2023/3540020

**Published:** 2023-03-09

**Authors:** Azam Sajjadi Nasab, Fatemeh Noorani, Zahra Paeizi, Leila Khani, Saba Banaei, Mohammad Sadeghi, Melika Shafeghat, Mahan Shafie, Mahsa Mayeli, The Alzheimer's Disease Neuroimaging Initiative (ADNI)

**Affiliations:** ^1^NeuroTRACT Association, Students' Scientific Research Center, Tehran University of Medical Sciences, Tehran, Iran; ^2^School of Medicine, Tehran University of Medical Sciences, Tehran, Iran; ^3^Alzheimer's Disease Neuroimaging Initiative, USA

## Abstract

**Introduction:**

While cerebrospinal fluid (CSF) core biomarkers have been considered diagnostic biomarkers for a long time, special attention has been recently dedicated to lipoproteins and metabolites that could be potentially associated with Alzheimer's disease (AD) neurodegeneration. Herein, we aimed to investigate the relationship between the levels of CSF core biomarkers including A*β*-42, TAU, and P-TAU and plasma lipoproteins and metabolites of patients with AD from the baseline cohort of the Alzheimer's Disease Neuroimaging Initiative (ADNI) database.

**Method:**

Using the ADNI database, fourteen subclasses of lipoproteins as well as a number of lipids and fatty acids and low-molecular metabolites including amino acids, ketone bodies, and glycolysis-related metabolites in blood samples were measured as potential noninvasive markers, and their association with the CSF core biomarkers was statistically investigated controlling for age and gender.

**Results:**

A total number of 251 AD subjects were included, among whom 71 subjects were negative for the *Apo-E ε*4 allele and 150 were positive. There was no significant difference between the two groups regarding cognitive assessments, CSF core biomarkers, and lipoproteins and metabolites except the level of A*β*-42 (*p* < 0.001) and phenylalanine (*p* = 0.049), which were higher in the negative group. CSF TAU and P-TAU were significantly correlated with medium and small HDL in the negative group, and with extremely large VLDL in the positive group. Our results also indicated significant correlations of metabolites including unsaturated fatty acids, glycerol, and leucine with CSF core biomarkers.

**Conclusion:**

Based on our findings, a number of lipoproteins and metabolites were associated with CSF core biomarkers of AD. These correlations showed some differences in *Apo-E ε*4 positive and negative groups, which reminds the role of *Apo-E* gene status in the pathophysiology of AD development. However, further research is warranted to explore the exact association of lipoproteins and other metabolites with AD core biomarkers and pathology.

## 1. Introduction

Alzheimer's disease (AD) is a progressive neurodegenerative dementia primarily characterized by memory loss and cognitive decline. Pathologically, different proteins including amyloid-*β* (A*β*) and tubulin-associated unit (TAU) play a key role in the initiation and progression of the disease with the formation of A*β* plaques in the brain cortex and deposition of phosphorylated-TAU (P-TAU) in the neurofibrillary tangles (1). Moreover, recent studies have emphasized the pivotal role of these biomarkers in the early diagnosis of AD (2).

There are various well-known genetic and nongenetic risk factors associated with AD, while evidence on the genetic aspect of the disease shows that the *Apo-E ε*4 allele is the strongest genetic risk factor associated with sporadic AD. Although it is not essential for the development of the disease, subjects with this allele are at a higher risk of developing the disease (3). Some studies suggested that Apo-E4 affects the accumulation of A*β* and P-TAU lesions; however, the exact role of Apo-E4 involvement in AD remains unknown (4, 5). Recently, some advances indicated the role of Apo-E in the other aspects of AD physiopathology including various plasma and cerebrospinal fluid (CSF) biomarkers (3).

Currently, in addition to the clinical presentation of the disease, the laboratory levels of three biomarkers in the CSF including A*β*-42, TAU, and P-TAU are one of the components of the diagnosis of AD, among which CSF A*β*-42 showed the highest accuracy with relatively high sensitivity and specificity. However, a variety of different CSF and plasma biomarkers have been recently suggested in the past few years, which could be utilized in the diagnosis of AD and the prediction of the disease progression (6). A number of major metabolite groups including lipids, amino acids, carbohydrates, vitamins, and even bile acids have a proposed role in AD development, which showed this potential to be utilized as biomarkers. Some recent studies have found the association between these metabolites and AD progression (7).

Lipid metabolism has been shown to be associated with AD pathogenesis. Various studies have already reported that higher levels of cholesterol may cause higher rates of A*β* deposition in the brain, while lower levels of cholesterol have been reported to be involved in AD pathogenesis via the nonamyloidogenic pathway. However, studies on lipids profile in patients with AD have produced inconsistent results, and the alterations of lipids and lipoproteins in AD are still controversial. Besides, a number of studies investigating peripheral biomarkers in AD have suggested preclinical biomarkers, which are associated with lipid metabolism (8). Notably, a systematic review on plasma lipids as biomarkers of AD indicated an association between plasma lipids including serum low-density lipoprotein (LDL), triglycerides (TG), and total cholesterol (TC), and AD development, proving that plasma lipids can be used as biomarkers for early diagnosis of AD (9). Several studies have investigated a variety of metabolites in association with AD. Studies on metabolomics have reported the altered concentrations of several amino acids including tryptophan, phenylalanine, histidine, and alanine in blood and urine, and CSF in AD (7). A recent study on metabolites associated with AD reported seven metabolites including amino acids and fatty acids (FAs) associated with executive function among AD patients. This study also suggested that FAs might be associated with cognitive impairment more complex than previously suspected (10).

Meanwhile, only a few studies have evaluated the association between lipoproteins and metabolites with previously known biomarkers. A recent systematic review and meta-analysis reviewed 24 articles on CSF A*β*, TAU, apolipoprotein, and other brain metabolites and concluded that N-acetyl aspartate and glutamate could be associated with the level of A*β* and TAU (11). Recent research also noted the association of serum Apo-B with CSF core biomarkers of AD and suggested that Apo-B could be a potential biomarker for early-stage AD (12). Hence, further studies are warranted to increase our understanding of alterations of plasma lipid profile in AD, specifically in association with previously known core biomarkers.

Herein, we aimed to investigate the relationship between the levels of well-established CSF biomarkers including A*β*-42, TAU, and P-TAU and plasma lipoproteins and metabolites of patients with AD from the baseline cohort of the Alzheimer's Disease Neuroimaging Initiative (ADNI) database, as we hypothesized that the level of these plasma lipoproteins and metabolites might be related to the disease development and serve as markers of the disease. We included various subclasses of lipoproteins, FAs, glycolysis-related metabolites, amino acids, and ketone bodies as potential biomarkers to investigate their associations with A*β*-42, TAU, and P-TAU controlling for age and gender in each negative and positive Apo-E4 groups.

## 2. Methods

### 2.1. Data and Participants

The data used in the preparation of this article were obtained from the ADNI database (http://adni.loni.usc.edu). The ADNI was launched in 2003 by the National Institute on Aging, the National Institute of Biomedical Imaging and Bioengineering, the US Food and Drug Administration, private pharmaceutical companies, and nonprofit organizations as a private partnership. The primary goal of ADNI has been to test whether serial magnetic resonance imaging (MRI), positron emission tomography (PET), other biological markers, and clinical and neuropsychological assessment can be combined to measure the progression of mild cognitive impairment (MCI) and early AD. Further information about ADNI can be found at http://adni.loni.usc.edu.

Inclusion and exclusion criteria for ADNI participants are detailed at http://adni.loni.usc.edu. Briefly, all participants included in the ADNI database were from 55 to 90 years old, had 6 years or more of education, spoke English or Spanish fluently, and were without any severe neurological diseases except AD. In ADNI, participants were classified as cognitively normal (CN), MCI, or AD based on criteria comprehensively discussed on the ADNI website. The AD group met the National Institute of Neurological and Communicative Disorders and Stroke/Alzheimer's Disease and Related Disorders Association (NINCDS-ADRDA) criteria for AD.

In this study, we included all patients with AD diagnosis for whom data including demographics, *Apo-E* gene status, cognitive assessments (Clinical Dementia Rating (CDR) Sum of Boxes and Alzheimer's disease assessment scale (ADAS)), CSF AD-related proteins (A*β*-42, TAU, and P-TAU), lipoproteins and metabolites data were available. Eventually, our study consisted of 221 AD subjects among whom 71 subjects were *Apo-E ε*4 negative and 150 subjects were *Apo-E ε*4 positive.

### 2.2. Lipoproteins and Metabolites

In this study, lipoproteins and metabolites data were extracted from the Nuclear Magnetic Resonance (NMR) analysis of human serum samples from the ADNI using the Nightingale Health platform, which is available in the ADNI database. Nightingale Health has developed an NMR-based blood biomarker analysis assay, quantifying over 220 metabolic biomarkers from a single blood sample. NMR and mass spectrometry have become key technologies in the metabolomics field. However, Nightingale's NMR platform has an advantage when compared to mass spectrometry approaches as it provides absolute concentrations of metabolic measures, rather than relative concentrations, which increases the interpretability of the biomarker data provided. Additional information regarding sample preparation and processing is available in detail at http://adni.loni.usc.edu.

In this study, data regarding lipoproteins, FAs, and various low-molecular metabolites including amino acids, ketone bodies, and gluconeogenesis-related metabolites, which were available in Nightingale's NMR analysis, were included. All included lipoproteins and metabolites are described in Supplementary Table 1. Briefly, 14 subclasses of lipoproteins, two apolipoproteins, 10 FAs, five glycolysis-related metabolites, nine amino acids, three ketone bodies, and two fluid balance metabolites (creatinine and albumin) were included in the final analysis.

### 2.3. CSF A*β*-42, TAU, and P-TAU

CSF A*β*-42, TAU, and P-TAU extracted from ADNI were analyzed by the electrochemiluminescence immunoassay (ECLIA) Elecsys which is currently under development by Roche Diagnostics and not commercially available yet. Roche Elecsys is a validated and fully automated immunoassay, which is utilized in conducting in vitro tests for the quantitative determination of the concentration of the mentioned proteins in human CSF. A detailed description of the biomarkers assessments is available on ADNI.

### 2.4. Statistical Analysis

Continuous variables were reported as mean ± standard deviation (SD) and compared between negative and positive Apo-E4 groups using the Mann–Whitney *U* test or independent-sample *t*-test based on the distribution of the data. Gender was compared between two groups using Pearson's chi-square test. To test the correlation between the levels of CSF A-*β*, TAU, and P-TAU and lipoproteins and metabolites, partial correlation tests adjusted for age and gender were performed in each negative and positive Apo-E4 group. All statistical analyses were also performed using SPSS version 26.0 (IBM SPSS Inc., Armonk, New York, USA) and a *p* value less than 0.05 was considered significant.

## 3. Results

### 3.1. Participants and Demographics

A total number of 251 subjects were included, among whom 71 subjects were negative for the *Apo-E ε*4 allele and 150 were positive.  Table 1 illustrates the demographics and clinical characteristics of participants in each group. The mean age in the negative Apo-E4 group was 76.45 ± 9.09 years, which was significantly higher than the positive group with a mean of 73.88 ± 7.75 (*p* = 0.019). However, there was no significant difference in gender distribution and mean education level between the two groups (*p* = 0.066 and 0.183, respectively). The two groups did not differ regarding cognitive assessments including CDR and ADAS scores (*p* = 0.501 and 0.381, respectively). Regarding CSF proteins, A*β*-42 was significantly higher in the negative group (*p* < 0.001). However, TAU and P-TAU levels did not show any significant difference between the two groups (*p* = 0.215 and 0.067, respectively). Moreover, all studied lipoproteins and metabolites did not yield any significant difference between the two groups, except phenylalanine which was slightly higher in the negative group (*p* = 0.049).

### 3.2. Correlations between CSF Proteins and Studied Lipoproteins and Metabolites

Significant and marginally significant correlations between the levels of CSF proteins and studied lipoproteins and metabolites adjusted for age and gender in each negative and positive Apo-E4 group are demonstrated in Table 2.

### 3.3. Lipoproteins

Among 14 studied lipoproteins, there were four lipoproteins that showed a significant correlation with at least one of CSF A*β*-42, TAU, and P-TAU. Extremely large very low-density lipoprotein (XXL VLDL) was significantly correlated with TAU (*p* = 0.031) and P-TAU (*p* = 0.033) in the positive group; however, it did not show any significant correlation with CSF proteins in the negative group. Large high-density lipoprotein (L HDL) showed a marginally significant correlation with P-TAU in the negative group (*p* = 0.091) without any significant result in the positive group. Medium high-density lipoprotein (M HDL) and small high-density lipoprotein (S HDL) showed a similar pattern and both were significantly correlated with TAU and P-TAU in the negative group (*p* = 0.006 and 0.004 for M HDL and *p* = 0.011 and 0.011 for S HDL, respectively) without any significant result in the other group. All other lipoproteins did not yield any significant correlation with CSF proteins in our analysis.

### 3.4. Apolipoproteins

Apo-A1 showed a significant correlation with P-TAU and a marginally significant correlation with TAU in the negative group (*p* = 0.029 and 0.063, respectively). Apo-B, however, was not correlated with any CSF proteins.

### 3.5. Fatty Acids

Docosahexaenoic acid (DHA) was marginally significantly correlated with A*β*-42 in the positive group (*p* = 0.058) without any significant correlation in the other group. Linoleic acid (LA) showed a marginally significant correlation with A*β*-42 but in the negative group (*p* = 0.099) without any significant correlation in the other group. The level of omega-3 FAs showed significant and marginally significant correlation with P-TAU and TAU in the positive group (*p* = 0.048 and 0.062, respectively), and the level of omega-6 FAs was marginally correlated with A*β*-42 in the negative group (*p* = 0.080). The level of polyunsaturated FA (PUFA) was marginally correlated with A*β*-42 in the negative group (*p* = 0.092), and the level of monounsaturated FA (MUFA) was correlated with A*β*-42 in the positive group (*p* = 0.099). Any other FAs showed no significant correlation with CSF biomarkers.

### 3.6. Glycolysis-Related Metabolites

Glycerol was the only glycolysis-related metabolite, which showed a significant correlation with CSF A*β*-42 in the negative Apo-E4 group (*p* = 0.037). All other glycolysis-related metabolites did not show any significant correlation with CSF proteins in our analysis.

### 3.7. Amino Acids

Among amino acids, only alanine, leucine, valine, and phenylalanine showed significant or marginally significant correlation with at least one of the CSF proteins with a relatively similar pattern. Alanine was marginally significantly correlated with A*β*-42 in the negative group (*p* = 0.060), leucine was significantly correlated with A*β*-42 in the negative group (*p* = 0.014), valine was marginally significantly correlated with A*β*-42 and P-TAU in the negative group (*p* = 0.056 and 0.096, respectively), and phenylalanine was marginally significantly correlated with A*β*-42 in the negative group (*p* = 0.066). Other amino acids did not show any significant correlation with CSF proteins in either group.

### 3.8. Fluid Balance Metabolites

Albumin showed a marginally significant correlation with TAU and P-TAU (*p* = 0.058 and 0.065, respectively); however, creatinine did not show any significant correlation with any CSF proteins.

## 4. Discussion

In this study, we investigated the correlations between subclasses of lipoproteins and metabolites, and CSF core biomarkers of AD separately in negative and positive Apo-E4 groups. There was no significant difference between the two groups regarding cognitive assessments and CSF core biomarkers except A*β*-42. Besides, lipoproteins and metabolites showed no difference between the two groups except phenylalanine. CSF TAU and P-TAU were significantly correlated with M HDL and S HDL in the negative group, and with XXL VLDL in the positive group. Our results also indicated associations of metabolites including unsaturated fatty acids, glycerol, and leucine with CSF core biomarkers.

It has been about three decades that the *Apo-E* gene continues to be the strongest genetic risk factor associated with sporadic AD despite several recent advances in AD genetic bases (3). Classically, the *Apo-E ε*4 allele has been suggested to be associated with A*β* accumulation at the top of the amyloid cascade; however, recent evidence has shown other pathophysiological processes for *Apo-E* alleles rather than A*β* metabolism (13). From a pathophysiological aspect, although hepatocytes are the main producer of Apo-E in the periphery, astrocytes and microglia are the main sources of Apo-E in the normal brain (14). In AD, however, a downregulation in Apo-E expression in reactive astrocytes and an upregulation in activated microglia have been observed (15). From animal models to human postmortem autopsy and A*β* PET studies, it has been shown that Apo-E4 promotes the deposition of A*β* and inhibits degradation of it in AD resulting in symptom development, while no direct interaction between Apo-E and TAU proteins has been introduced. Indeed, the indirect impact of Apo-E on microglia and astrocytes could ultimately lead to TAU-induced neurodegeneration (3). Similarly, in our study, CSF A*β* showed a significant difference between the Apo-E4 negative and positive groups, while the levels of TAU and P-TAU did not differ. Besides, the *Apo-E* genotype could mainly affect the age of onset of cognitive impairment. Similarly in our sample, patients positive for *Apo-E ε*4 allele had significantly lower age; however, we found no significant difference between the two groups in AD cognitive assessments (i.e., CDR and ADAS). Apo-E participates in the distribution and metabolism of lipids among various tissues of the body (16). Previous studies on AD have reported alterations in various lipoproteins in association with the *Apo-E* gene and indicated that Apo-E4 could increase circulating cholesterol levels, primarily by raising LDL levels (17, 18). In our study, however, none of the fourteen studied lipoproteins showed a significant difference between Apo-E4 positive and negative groups. Nevertheless, current evidence on the lipid profile among *Apo-E ε*4 carriers is still inconsistent and controversial (4, 5, 19).

Although several previous studies have shown a link between lipid metabolism and AD, the exact pathogenesis is still unclear. Current evidence suggested that increased serum levels of TC, TG, and LDL and decreased HDL are associated with increased deposition of A*β* plaques which could result in AD. However, the alterations in the level of serum lipoproteins, different lipids, and related metabolites remain controversial. A recent systematic review and meta-analysis on the impact of cholesterol on the risk of AD concluded that only LDL level is associated with AD (20). Similarly, another systematic review and meta-analysis reported that elevated concentration of LDL could be a potential risk factor for AD and this association is strong in patients in their 60s, which fades with advancing age (21). Other studies emphasized the other lipids and lipoproteins and reported lower serum level of HDL (22) and elevated serum level of TC (23) in the cases with AD. On contrary, some studies reported that late-life hypercholesterolemia might slow cognitive decline, particularly in combination with other cerebrovascular risk factors, possibly due to enhanced cerebral perfusion (24).

Nevertheless, the association between plasma lipid profile and previously known core biomarkers has been less investigated through the literature. Our results indicated that among participants in the negative Apo-E4 group, CSF TAU and P-TAU proteins were significantly correlated with only M HDL and S HDL, while no significant correlation was yielded for other lipoproteins. In the positive Apo-E4 group, however, a significant correlation was observed between CSF TAU and P-TAU and XXL VLDL. A previous study on plasma A*β* and lipoproteins showed that A*β* was associated with plasma lipoproteins, especially those enriched with triglyceride including chylomicrons, VLDL, and intermediate-density lipoprotein (IDL) (25). A study on postmortem AD patients also found a significant correlation of A*β*-42 in the cerebral cortex with serum TC, LDL, and Apo-B independent of the *Apo-E* genotype (26). A recent study on blood-based biomarkers for age-related cognitive impairment demonstrated correlations between serum levels of lipoproteins and core biomarkers (A*β*-42 and TAU). In this study, TAU showed positive correlations with cholesterol ratio (TC/HDL), TG, and LDL and a negative correlation with HDL. Furthermore, A*β*-42 showed positive correlations with TG, LDL, and cholesterol ratio and negative correlations with HDL and TC (22). In our analysis, Apo-A1 also showed a correlation with TAU and P-TAU in the negative Apo-E4 group, while Apo-B did not yield any significant result. Conversely, previous studies have shown that AD patients had an increased level of Apo-B compared to healthy controls, which showed a significant positive correlation with cortical A*β* (26).

Our results also indicated significant correlations between a number of blood-based FAs and amino acids, and CSF core biomarkers. Among studied FAs, LA, omega-6 FAs, and PUFA showed links with A*β*-42 in the negative group and DHA, omega-3 FAs, and MUFA showed links with A*β*-42 in the negative group. Our findings were on the same line with previously known evidence on serum-based metabolomic studies. As an example, a review on metabolites associated with AD pathology reported imbalances in phospholipid composition after reviewing over 10 studies (27). There is increasing evidence in favor of the fact that elevated saturated FAs could have negative effects on age-related cognitive decline; however, on contrary, a higher level of MUFA and PUFA intake, particularly omega-3 FAs, has been suggested to be associated with a lower risk of cognitive decline (28). Evidence has suggested that omega-3 FAs may act as a possible protective factor in AD (29, 30). Moreover, some studies have pointed to the role of omega-3 FA supplements in preventing AD, specifically Apo-E4-associated AD (31, 32). However, a recent randomized controlled trial reported that omega-3 PUFA supplements did not reduce the cognitive, functional, and blood-based biochemical profile of patients with AD (33). Regarding omega-6 FAs, a systematic review concluded that there is an association between the omega-6/omega-3 ratio, AD development, and cognitive decline (34). Despite controversial findings, these data support the idea that MUFA and PUFA may play a role in AD and cognitive decline. However, the association between these FAs and A*β* and TAU pathologies has been less known. A recent animal study reported that omega-3 PUFAs could promote A*β* clearance from the brain to blood, which could be an effective strategy to prevent the progression of AD (35). A recent review also concluded that omega-3 PUFAs reduce cerebral A*β* deposition (36). A comparative study on the plasma lipid profile of AD and MCI indicated alterations in a variety of FA-related metabolites including saturated and unsaturated FAs compared to healthy controls (37).

Regarding glycolysis-related metabolites, we only found a significant association of glycerol with A*β*-42 in the negative Apo-E4 group, while all other metabolites did not yield any correlation with A*β*-42, TAU, and P-TAU. Our analysis of amino acids showed A*β*-42 was significantly correlated with leucine and marginally with alanine, valine, and phenylalanine, whereas TAU was only partially correlated with valine, all in the negative group. A previous study has reported a significant alteration in a number of metabolites related to carbohydrate metabolism and tricarboxylic cycle among AD and MCI patients compared to healthy controls. In this study, contrary to our findings, glutamine, glutamate, and proline showed a significant difference between AD and the control group. However, after a subgroup analysis in Apo-E4 positive and negative groups, no difference in plasma metabolic profile was observed (37). A number of other studies also have investigated the differences in amino acids in different body fluid samples among patients with AD; however, the exact association of these metabolites with CSF core biomarkers is less reported (27, 38).

It is essential to interpret our findings considering a number of limitations. First, although we included all AD subjects for whom lipoproteins and metabolites data were available from ADNI database, a larger sample size could affect the final findings. Additionally, this study was an observational cross-sectional study with known intrinsic restrictions for interpreting data; therefore, a longitudinal study could expand our knowledge regarding the role of lipoproteins and metabolites in AD. Furthermore, several metabolites were not available in our extracted data from ADNI, while they could be potentially investigated as a biomarker of AD.

In conclusion, based on our findings in this study, lipoproteins and metabolites were associated with CSF core biomarkers of AD. These correlations showed some differences in Apo-E4 positive and negative groups, which reminds the role of *Apo-E* gene status in the pathophysiology of AD development. However, further research is warranted to explore the exact association of lipoproteins and other metabolites with AD previously known biomarkers and pathology.

## Figures and Tables

**Table 1:** Demographics and clinical characteristics of patients.

Measure	*Apo-E* status		*p* value
	Negative	Positive	
	*N* = 71	*N* = 150	
Age (years)	76.45 ± 9.09	73.88 ± 7.75	0.019
Gender			
Male	42	86	0.066
Female	29	64	
Education (years)	15.76 ± 2.91	15.23 ± 2.92	0.183
CDR	4.51 ± 1.59	4.47 ± 1.75	0.501
ADAS	30.48 ± 8.24	29.61 ± 8.10	0.381

CSF proteins			
A*β*-42	854.81 ± 412.66	586.47 ± 218.54	<0.001
TAU	359.80 ± 163.94	375.09 ± 135.07	0.215
P-TAU	35.09 ± 17.87	37.82 ± 14.76	0.067

Lipoproteins and metabolites^∗^			
Phenylalanine	0.069 ± 0.011	0.067 ± 0.010	0.049

^∗^Only lipoproteins and metabolites with significant or marginally significant differences mentioned in the table. CDR: clinical dementia rating, ADAS: Alzheimer's disease assessment scale, CSF: cerebrospinal fluid.

**Table 2:** Significant correlations between CSF core biomarkers and studied lipoproteins and metabolites controlled for age and gender in *Apo-E* groups.

Variable	*Apo-E* status					
	Negative			Positive		
	A*β*-42	TAU	P-TAU	A*β*-42	TAU	P-TAU
Lipoproteins						
XXL VLDL						
Rho	0.186	-0.003	-0.033	-0.022	-0.210	-0.208
*p* value	0.196	0.986	0.821	0.819	**0.031**	**0.033**
L HDL						
Rho	-0.175	0.201	0.241	-0.027	0.081	0.049
*p* value	0.225	0.161	**0.091**	0.780	0.407	0.619
M HDL						
Rho	-0.132	0.386	0.400	-0.082	0.122	0.115
*p* value	0.360	**0.006**	**0.004**	0.405	0.213	0.242
S HDL						
Rho	-0.060	0.385	0.355	-0.072	0.068	0.089
*p* value	0.680	**0.011**	**0.011**	0.461	0.489	0.367
Apolipoproteins						
Apo-A1						
Rho	-0.158	.225	0.263	0.003	-0.006	-0.036
*p* value	0.195	**0.063**	**0.029**	0.970	0.942	0.662
Fatty acids						
DHA						
Rho	-0.136	0.095	0.154	-0.157	-0.075	-0.110
*p* value	0.266	0.437	0.206	**0.058**	0.369	0.184
LA						
Rho	-0.200	0.032	0.090	-0.021	-0.043	-0.065
*p* value	**0.099**	0.794	0.460	0.804	0.605	0.437
Omega-3						
Rho	-0.119	0.094	0.146	-0.155	-0.133	-0.164
*p* value	0.332	0.445	0.232	**0.062**	0.108	**0.048**
Omega-6						
Rho	-0.212	0.083	0.145	-0.047	-0.039	-0.062
*p* value	**0.080**	0.496	0.235	0.573	0.643	0.453
PUFA						
Rho	-0.204	0.088	0.151	-0.067	-0.056	-0.082
*p* value	**0.092**	0.470	0.216	0.421	0.501	0.323
MUFA						
Rho	-0.083	0.068	0.093	-0.136	0.010	0.007
*p* value	0.500	0.579	0.449	**0.099**	0.903	0.932
Glycolysis-related metabolites						
Glycerol						
Rho	0.262	-0.013	-0.048	-0.012	-0.058	-0.046
*p* value	**0.037**	0.918	0.706	0.891	0.488	0.582
Amino acids						
Alanine						
Rho	0.237	-0.116	-0.159	-0.023	0.043	0.032
*p* value	**0.060**	0.360	0.209	0.781	0.609	0.703
Leucine						
Rho	0.305	-0.132	-0.181	0.017	-0.049	-0.024
*p* value	**0.014**	0.297	0.153	0.841	0.560	0.776
Valine						
Rho	0.241	-0.160	-0.210	0.056	-0.035	-0.013
*p* value	**0.056**	0.207	**0.096**	0.509	0.677	0.877
Phenylalanine						
Rho	0.231	-0.030	-0.026	-0.096	-0.046	-0.054
*p* value	**0.066**	0.811	0.835	0.254	0.587	0.525
Fluid balance metabolites						
Albumin						
Rho	-0.001	0.102	0.131	-0.077	-0.159	-0.155
*p* value	0.997	0.422	0.303	0.362	**0.058**	**0.065**

XXL VLDL: extremely large very low-density lipoprotein, L HDL: large high-density lipoprotein, M HDL: medium high-density lipoprotein, S HDL: small high-density lipoprotein, DHA: docosahexaenoic acid, LA: linoleic acid, PUFA: polyunsaturated fatty acids, MUFA: monounsaturated fatty acids.

## Data Availability

The data used in this research was obtained from ADNI and is available with permission to all researchers. Data used in the preparation of this article were obtained from the Alzheimer's Disease Neuroimaging Initiative (ADNI) database (http://adni.loni.usc.edu). As such, the investigators within the ADNI contributed to the design and implementation of ADNI and/or provided data but did not participate in the analysis or writing of this report. A complete listing of ADNI investigators can be found at: http://adni.loni.usc.edu/wp-content/uploads/how_to_apply/ADNI_Acknowledgement_List.pdf.

## Supplementary Materials

Supplementary Materials Supplementary Table 1: studied lipoproteins and metabolites.
