# Supplementary material for: A Comprehensive Investigation of the Potential Role of Lipoproteins and Metabolite Profile as Biomarkers of Alzheimer's Disease Compared to the Known CSF Biomarkers
Source: Int J Alzheimers Dis. 2023 Mar 9;2023:3540020. doi: 10.1155/2023/3540020 (PMC10019964; doi:10.1155/2023/3540020)
Supplement: Supplementary Materials — Supplementary Table 1: studied lipoproteins and metabolites. [file 3540020.f1.docx]

**Supplementary Table 1. Studied lipoproteins and metabolites**

| **Variable** | **Definition** |
| --- | --- |
| **CSF AD-related Proteins** | |
| **Aβ-42** | Amyloid beta |
| **TAU** | Tubulin associated unit protein |
| **P-TAU** | Phosphorylated tubulin associated unit |
| **Lipoproteins** | |
| **XXL VLDL** | Concentration of chylomicrons and extremely large VLDL particles |
| **XL VLDL** | Concentration of very large VLDL particles |
| **L VLDL** | Concentration of large VLDL particles |
| **M VLDL** | Concentration of medium VLDL particles |
| **S VLDL** | Concentration of small VLDL particles |
| **XS VLDL** | Concentration of very small VLDL particles |
| **IDL** | Concentration of IDL particles |
| **L LDL** | Concentration of large LDL particles |
| **M LDL** | Concentration of medium LDL particles |
| **S LDL** | Concentration of small LDL particles |
| **XL HDL** | Concentration of very large HDL particles |
| **L HDL** | Concentration of large HDL particles |
| **M HDL** | Concentration of medium HDL particles |
| **S HDL** | Concentration of small HDL particles |
| **Apolipoproteins** | |
| **Apo-A1** | Apolipoprotein A-I |
| **Apo-B** | Apolipoprotein B |
| **Lipids and Fatty acids** | |
| **Total Cholesterol** | Serum total cholesterol |
| **Total Triglyceride** | Serum total triglycerides |
| **Tot FA** | Total fatty acids |
| **DHA** | 22:6, docosahexaenoic acid |
| **LA** | 18:2, linoleic acid |
| **Omega-3** | Omega-3 fatty acids |
| **Omega-6** | Omega-6 fatty acids |
| **PUFA** | Polyunsaturated fatty acids |
| **MUFA** | Monounsaturated fatty acids; 16:1, 18:1 |
| **SFA** | Saturated fatty acids |
| **Glycolysis-related metabolites** | |
| **Glucose** | Level of Glucose |
| **Lactate** | Level of Lactate |
| **Pyruvate** | Level of Pyruvate |
| **Citrate** | Level of Citrate |
| **Glycerol** | Level of Glycerol |
| **Amino acids** | |
| **Alanine** | Level of Alanine |
| **Glutamine** | Level of Glutamine |
| **Glycine** | Level of Glycine |
| **Histamine** | Level of Histidine |
| **Isoleucine** | Level of Isoleucine |
| **Leucine** | Level of Leucine |
| **Valine** | Level of Valine |
| **Phenylalanine** | Level of Phenylalanine |
| **Tyrosine** | Level of Tyrosine |
| **Ketone bodies** | |
| **Acetate** | Level of Acetate |
| **Acetoacetate** | Level of Acetoacetate |
| **bOHBut** | 3-hydroxybutyrate |
| **Fluid balance metabolites** | |
| **Creatinine** | Level of Creatinine |
| **Albumin** | Level of Albumin |
